# Genetic Diversity and Population Structure Analysis of the USDA Olive Germplasm Using Genotyping-By-Sequencing (GBS)

**DOI:** 10.3390/genes12122007

**Published:** 2021-12-17

**Authors:** A. S. M. Faridul Islam, Dean Sanders, Amit Kumar Mishra, Vijay Joshi

**Affiliations:** 1 Texas A&M AgriLife Research and Extension Center, Uvalde, TX 78801, USA; farid-bge@ag.tamu.edu; 2 Bioinformatics Resource Center, University of Wisconsin–Madison, Madison, WI 53706, USA; dmsanders@wisc.edu; 3 Department of Botany, School of Life Sciences, Mizoram University, Aizawl 796004, Mizoram, India; amit.bhu.bot@gmail.com; 4 Department of Horticultural Sciences, Texas A&M University, College Station, TX 77843, USA

**Keywords:** genotyping-by-sequencing (GBS), SNP, population structure, AMOVA

## Abstract

Olives are one of the most important fruit and woody oil trees cultivated in many parts of the world. Olive oil is a critical component of the Mediterranean diet due to its importance in heart health. Olives are believed to have been brought to the United States from the Mediterranean countries in the 18th century. Despite the increase in demand and production areas, only a few selected olive varieties are grown in most traditional or new growing regions in the US. By understanding the genetic background, new sources of genetic diversity can be incorporated into the olive breeding programs to develop regionally adapted varieties for the US market. This study aimed to explore the genetic diversity and population structure of 90 olive accessions from the USDA repository along with six popular varieties using genotyping-by-sequencing (GBS)-generated SNP markers. After quality filtering, 54,075 SNP markers were retained for the genetic diversity analysis. The average gene diversity (GD) and polymorphic information content (PIC) values of the SNPs were 0.244 and 0.206, respectively, indicating a moderate genetic diversity for the US olive germplasm evaluated in this study. The structure analysis showed that the USDA collection was distributed across seven subpopulations; 63% of the accessions were grouped into an identifiable subpopulation. The phylogenetic and principal coordinate analysis (PCoA) showed that the subpopulations did not align with the geographical origins or climatic zones. An analysis of the molecular variance revealed that the major genetic variation sources were within populations. These findings provide critical information for future olive breeding programs to select genetically distant parents and facilitate future gene identification using genome-wide association studies (GWAS) or a marker-assisted selection (MAS) to develop varieties suited to production in the US.

## 1. Introduction

Olives (*Olea europaea* L.) are one of the economically important fruit and oil trees contributing to the Mediterranean food diet. Often referred to as ‘liquid gold’ [1], olive oil is a rich source of functional compounds such as hydroxytyrosol, oleuropein, and monounsaturated fatty acids beneficial to human health [2]. Several therapeutic studies have confirmed the utility of olive oil in alleviating the impacts of cardiovascular disease, obesity, metabolic syndrome, type 2 diabetes, and hypertension [3,4,5]. The olive is believed to have been domesticated in the Mediterranean basin about 6000 years ago, subsequently spreading through the Mediterranean countries [3]. Although most commercial olive production is confined to Mediterranean countries, more than 40 countries grow olives including Argentina, the United States (USA), Australia, Chile, and China [3,6]. California is the central oil-producing state in the USA, yielding 67,000 tons of olives at a value of USD 57,909 million [7]. Even though the US produces less than 1% of the world’s olives, it represents the third largest national market for olive oil globally, the most significant market outside the European community. Olives were believed to have been first introduced into the US by Spanish Franciscan missionaries in the late 18th century [8].

Genetic diversity is essential for any crop improvement program. Genetic improvements require in-depth screening to understand the nature and extent of the diversity within the germplasm [9]. The National Clonal Germplasm Repository (NCGR) at the University of California-Davis (UC-Davis), one of the clonal gene banks under the National Plant Germplasm System of the USDA-ARS, holds a collection of olive accessions collected from all over the world. This collection represents a valuable tool for population and evolutionary genetic studies of olives and a source of material for breeding purposes. Correctly identifying olive cultivars is challenging due to the high degree of kinship, clonal variation, mixtures with international cultivars, and exchange of plant material over the centuries. The complex distribution pattern resulted in homonymies and synonymies as well as naming errors of cultivars [10,11,12]. A detailed molecular evaluation of the olive accessions in the US repository would provide insights into the amount and organization of genetic diversity and relationships within and among different accessions. Such studies would enhance the value of regionally adapted germplasm and allow for a better utilization and management of regional challenges such as abiotic stresses (e.g., freezing, drought, and nutrient deficiencies), disease resistance (for example, cotton root rot), and oil quality traits. It is imperative to examine the genetic variation and population structure of the olive germplasm to manage the gene pool effectively, help understand the effect of domestication on genetic diversity [10], and aid in the development of new cultivars.

Several morphological traits as well as biochemical and molecular markers have been used to characterize olive germplasm resources [13]. Various types of molecular markers such as random amplified polymorphism DNA (RAPD), amplified fragment length polymorphism (AFLP), sequence-related amplified polymorphism (SRAP), simple sequence repeat (SSR), inter-simple sequence repeat (ISSR), and single-nucleotide polymorphism (SNP) have been used for genetic studies and food traceability in olives has been extensively reviewed [5,12]. However, SNPs have become a marker of choice for various genetic studies due to their unique features such as their availability throughout the genome, unbiased distribution, biallelic nature, stability (e.g., a low mutation rate), and automation in next-generation sequencing (NGS) techniques [3,10,14,15]. The use of molecular markers facilitates an understanding of the parentage in the US olive collection. In addition, the use of SNP markers allows the study of the genetic correlations among the phenotypes of interest and their heritability as well as providing an estimation of the breeding values. Such studies have already been performed in long-lived perennial plants such as almonds, apricots, and apples [14,15,16,17,18].

Genotyping-By-Sequencing (GBS) is an NGS-based genome-wide SNP discovery and genotyping technique with cost- and time-effective characteristics [19]. It can sequence multiple samples simultaneously by using NGS libraries made with methylation-sensitive restriction enzymes, avoiding genome complexities whilst better sequencing lower copy genic regions in the plant genome [11]. GBS can be applied to any species with or without a reference genome, making it the most popular approach for SNP identification [3,10]. So far, this technology has been used to study genetic characterization, association mapping, QTL mapping, and genomic selection in major crops such as wheat [20], barley [21], maize [22], and rice [23,24] as well as fruit crops such as citrus [25], peach [26], apple [27] and oil palm [28]. GBS-generated SNP markers have been utilized in olives to construct high-density genetic linkage maps, a genetic diversity analysis of the germplasm of European and Mediterranean olives, and genome-wide association mapping (GWAS) [3,5,10,11]. Aside from a single study that used microsatellite markers to examine the genetic diversity within olive accessions maintained in the US NCGR collection [29], limited attempts have been made to characterize the olive accessions in the US repository.

In the present study, we used GBS technology to genotype a collection of olive accessions assembled from the USDA-ARS National Plant Germplasm System (NPGS). The objectives of this study were to: (1) generate SNP markers by GBS technology and evaluate their characteristics; (2) determine the population structure of the USDA germplasm collection; and (3) measure the genetic relationships and sources of the genetic variations. The knowledge of the genetic diversity and relationships within and among the accessions in the US olive repository would serve as a resource for effective conservation, management, and utilization of these accessions as well as developing superior cultivars that fulfil the needs of the US market.

## 2. Materials and Methods

### 2.1. Plant Materials

The study comprised 90 olive accessions obtained from the National Clonal Germplasm Repository (NCGR) at the University of California-Davis (UC-Davis) along with samples of 6 regionally popular olive varieties. For efficient rooting, the basal end of the cuttings was dipped in 1000 ppm indole butyric acid [30] for 10 s. After the IBA treatment, the cuttings were inserted in 2.5 × 14 inch Deepot tree pots containing perlite and kept under mist (80 to 90% relative humidity) at the Texas A&M AgriLife Research and Extension Center, Uvalde, TX, USA. The intermittent mist system was operated as needed to maintain uniform moisture around the cuttings. The olive accessions originated from 18 countries. The cuttings with newly sprouted leaves were transferred to new pots for the subsequent management. The 18 countries were categorized into 5 major climatic zones based on the Köppen Climate Classification [31] as tropical, dry, temperate, continental, and polar (Table 1). According to this classification, most samples (69 out of 96 accessions) belonged to the temperate climatic zone, followed by the dry zone (16), tropical zone (3), and the continental zone (2). The six olive accessions with no origin information were considered to be of an unknown origin.

### 2.2. DNA Extraction and Genotyping-By-Sequencing (GBS) Procedures

Leaf samples were collected from the different accessions in 2 mL centrifuge tubes and flash-frozen in liquid nitrogen. The frozen leaf tissue was homogenized to a fine powder in a Harbil model 5G-HD paint shaker (Harbil, Wheeling, IL, USA) using 3 mm Demag stainless steel balls (Abbott Ball Company, West Hartford, CT, USA). The total DNA was extracted using a DNeasy^®^ Plant Mini Kit (QIAGEN Sciences, Germantown, MD, USA) as per the manufacturer’s protocol and treated with RNase A. The purity of the DNA was analyzed using a NanoPhotometer spectrophotometer (IMPLEN, Westlake Village, CA, USA). An ApeKI restriction enzyme was used to construct the DNA libraries for the GBS. The library construction and sequencing by NovaSeq 6000 (Illumina, San Diego, CA, USA) were performed in the Bioinformatics Resource Center, University of Wisconsin–Madison. For the sequence analysis, low-quality reads and adapter sequences were removed from the raw fastq files using computational pipelines developed at the Bioinformatics Resource Center (BRC) at the University of Wisconsin–Madison (https://www.biotech.wisc.edu/, accessed on 23 February 2021) using a trimming software, Skewer [32]. The raw GBS sequences were processed using a standard TASSEL-GBS pipeline [33]. The details of the TASSEL-GBS Pipeline Version 2 (https://tassel.bitbucket.io/, accessed on 23 February 2021) are shown in Supplementary Figure S1.

### 2.3. Data Analysis

#### 2.3.1. SNP Discovery

The raw reads were quality trimmed to remove the adapters and low-quality bases (Phred ≥ 20) using Skewer software [32]. Once the quality raw fastq files were generated, the TASSEL Version 2 GBS pipeline was implemented to conduct the GBS analysis. In brief, a unique tag database was created by a GBSSeqtoTagDBPlugin that took quality controlled raw fastq files as the input data and then converted them into fastq files by the TagExportToFastqPlugin for the next alignment step. Bowtie 2 software was then used to align the exported tags against the *Olea europaea var. europaea* L. reference genome [34] and generate a sequence alignment map file (SAM) [35]. The SAM files were utilized by a SamToGBSdbPlugin to input the mapped genomic coordinates of each tag into the TASSEL database. The SNPs were identified using the aligned tags that were positioned at the same genomic coordinates using the DiscoverySNPCallerPluginV2, which required a MAF > 0.01 and a minimum locus coverage in all taxa of 10% (0.1). In the end, 349,851 unfiltered SNPs were discovered in the GBS analysis.

#### 2.3.2. SNP Marker Properties

To conduct the genetic diversity analysis, 54,075 biallelic SNP markers were finally selected based on the following filtering criteria using TASSEL 5 [36]: missingness rate < 0.5, minor allele frequency (MAF) > 0.05, and heterozygosity < 0.1.

The summary statistics including the minor allele frequency (MAF), gene diversity (GD), and polymorphic information content (PIC) for all SNP markers were calculated using the snpReady package in [37].

#### 2.3.3. Population Molecular Characterization

A model-based (Bayesian) method implemented in STRUCTURE 2.3.4 software [38] was used to infer the most probable number of clusters or subpopulations in our germplasm. The admixture model and correlated allele frequency were used to run five independent runs for each K ranging from 1 to 10 to assign a genotype into a particular subpopulation. For each run, 10,000 and 50,000 replications were used for the burn-in time and Markov Chain Monte Carlo (MCMC), respectively. The result of the STRUCTURE software was then submitted to CLUMPAK [39] to determine the best K by using ΔK values following the method of Evanno et al. [40]. A principal coordinate analysis (PCoA) was performed based on the Euclidean distance method to determine the overall genetic difference among the accessions. Both studies were conducted in the adegenet R package [30]. In addition, an unrooted phylogeny tree using the neighbor-joining method was constructed using MEGA7 [41].

An analysis of molecular variance (AMOVA) was carried out to determine the sources of the genetic variance within and among the populations detected by the STRUCTURE software. The Poppr R package [42] was used to calculate the AMOVA using the Euclidean genetic distance method with 999 permutations to declare the significance of a particular genetic variance. Furthermore, the nucleotide diversity per site and fixation index (Fst, Weir, and Cockerham’s 1984) were calculated to measure the genetic diversity within and between populations, respectively, using VCFtools [43].

## 3. Results

### 3.1. Characterization and Distribution of the GBS-Generated SNPs in the Olive Genomes

**Figure 1:**
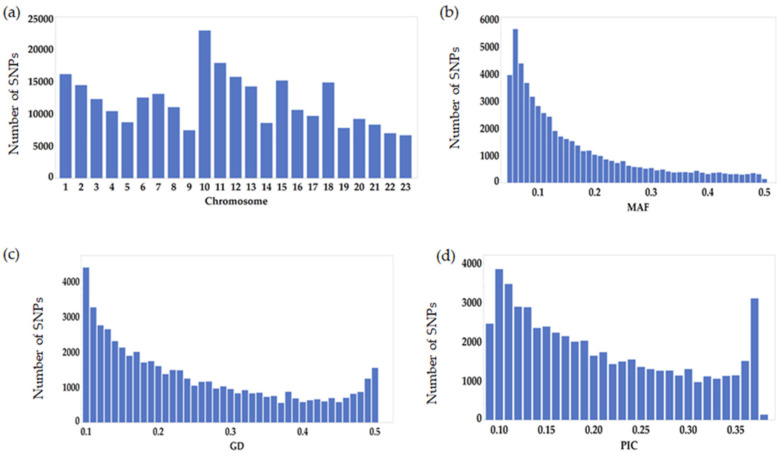
GBS-generated SNP marker characterization. (**a**) Number of SNPs per chromosome; (**b**) minor allele frequency (MAF); (**c**) gene diversity (GD); (**d**) polymorphic information content (PIC).

A total of 96 olives accessions were sequenced and genotyped using GBS. After filtering out the raw reads, the total demultiplexed reads for all the genotypes were 418.78 M with the average reads per accession being 4.36 M. The lowest and highest number of reads was 0.21 M and 11.35 M, respectively (Table S2). After processing the raw reads via the TASSEL-GBS pipeline and applying VCF filtering control thresholds, we were left with a subselected set of 54,075 SNPs. The dataset of 54,075 SNPs was then used for a further genetic diversity analysis. These SNPs were mapped onto 23 chromosomes along with 466 scaffolds. The highest and lowest SNPs mapped per chromosome were 22,870 and 6644 on chromosome 10 and chromosome 23, respectively, with an average of 11,906.22 SNP/chromosome (Figure 1a). On average, 163.11 SNPs were mapped to 466 scaffolds ranging from 1 to 2083 SNPs. Among the 54,075 SNPs, the transitions were more frequent (59%, 31,885 SNPs) than the transversions (41%, 22,190 SNPs) with an overall ratio of 1.44. The C/T transition had the highest frequency (30%) and the C/G transversion had the lowest (7%). The frequency of the two transition types was similar (A/G 29%, C/T 30%). The highest frequency among the transversions was found at A/T (14%). Two transversion SNP types (A/C and G/T) had the same frequency (10%) (Table 2).

### 3.2. Characterization of the SNP Markers

A total of 54,075 SNP markers were selected, satisfying the filtering criteria mentioned in the Method section, to conduct a genetic diversity analysis of the 96 olive germplasm used in this study. To explain the total variability of each marker, the minor allele frequency (MAF), gene diversity (GD), and polymorphic information content (PIC) were used. Although markers with a MAF < 0.05 were removed, the average MAF value was 0.160 with a minimum of 0.05 and a maximum of 0.50. About half (~44%) of the total markers (23,610 out of 54,075 markers) had a MAF less than or equal to 0.1 (Figure 1b). The mean gene diversity value was 0.244 with a maximum of 0.10 for 4389 markers and a minimum of 0.50 for 1546 markers (Figure 1c). The polymorphism information content (PIC) values ranged from 0.09 to 0.38 with an average of 0.21 (Figure 1d). Although 2451 SNPs had the lowest PIC value, 33% SNPs were found with a PIC value of half of its maximum theoretical PIC value (0.5); i.e., ≥0.25.

### 3.3. Characterization of the Population and the Genetic Relationships

#### 3.3.1. Structure Analysis and the Genetic Relationships

**Figure 2:**
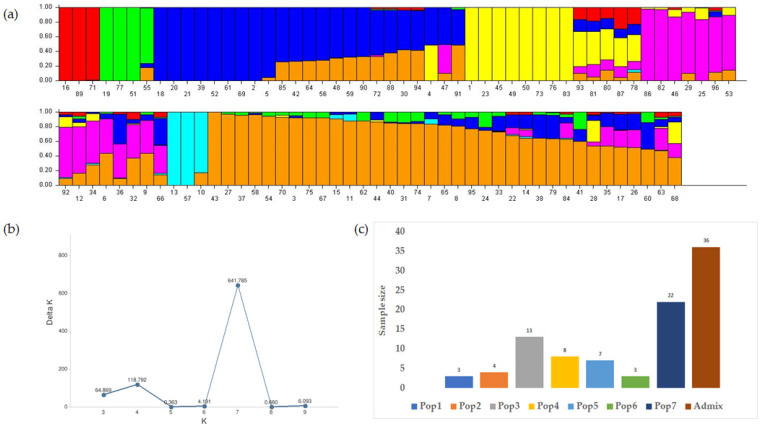
Population structure for 96 olive accessions from the USDA core collection using 54,075 SNP markers. (**a**) Subpopulation grouping inferred by the STRUCTURE software indicated in seven different colors. The y-axis values indicate the probability of the population. (**b**) Evanno test for the ideal population number using LnP(D)-derived ΔK from 2 to 10. At a value of K = 7, it reaches its highest value, indicating the most probable subpopulations in the germplasm. (**c**) Olive accession number based on the populations identified by the STRUCTURE software.

To understand the pattern of the genetic structure, a Bayesian clustering analysis in STRUCTURE was performed (Figure 2a). A population structure analysis was conducted using K values ranging from 2 to 10 with an admixture model and five independent runs for each K value were performed. An Evanno test was then performed to determine the log-likelihood (LnP(D)) values and ΔK between each K number. From the test, the top ΔK peak was found at K = 7, indicating that the US olive germplasm could be grouped into seven subpopulations with admixture accessions (Figure 2b). With a membership probability threshold of 0.70, a total of 60 olive accessions (63%) were grouped into one of seven subpopulations and the remaining 36 accessions were considered to be an admixture group (Figure 2a). The highest number of accessions that were grouped into a particular subpopulation (Pop7) was 22, followed by 13, 8, and 7 accessions clustered into 3 different subpopulations; Pop3, Pop4, and Pop5, respectively. From the remaining groups, two were composed of three accessions (Pop1 and Pop6), and one (Pop2) had four accessions (Table S1). In terms of the proportion of genotypes per climatic zone and distribution across the seven populations, 39, 20, and 14% of the temperate accessions were grouped into admixture, Pop7, and Pop3. In contrast, 38 and 19% of the dry climatic zone accessions were clustered into Pop7 and Pop3, respectively. The accessions from the continental zone were not grouped into any specific subpopulation outside the admixture group. Similarly, 67% of the tropical accessions did not belong to any subpopulation and the remaining 33% belonged to Pop2 (Figure 2c).

**Figure 3:**
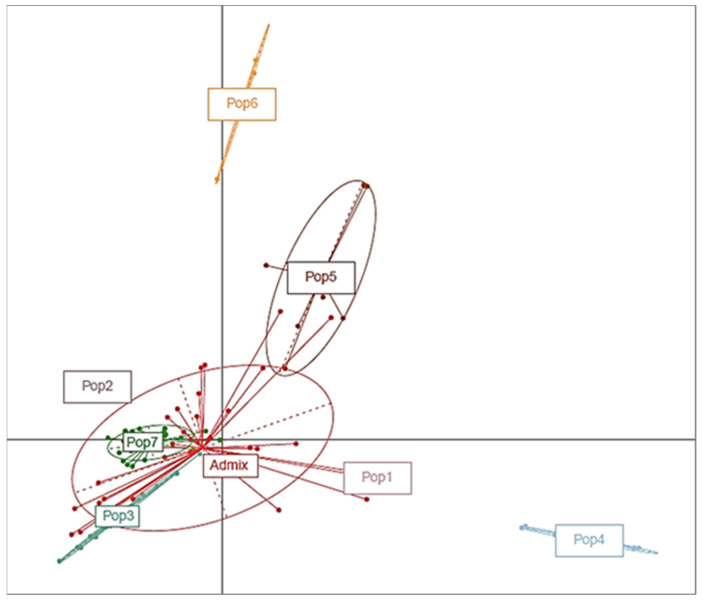
Principal coordinate analysis (PCoA). The analysis depicts the overall genetic diversity of the germplasm for 96 olive accessions using 54,075 SNP markers.

The principal coordinate analysis (PCoA) (Figure 3) agreed with the relationships revealed by the structure analysis. The PCoA based on the SNPs revealed seven clusters of the 96 accessions. Among the seven clusters, Pop1, Pop4, Pop5, and Pop6 were clustered distinctively from the remaining groups, indicating a genetically distinct relationship from the other groups. In contrast, Pop2, Pop3, and Pop7 were clustered into the same ellipsis, suggesting a close genetic relationship among those accessions (Figure 3).

**Figure 4:**
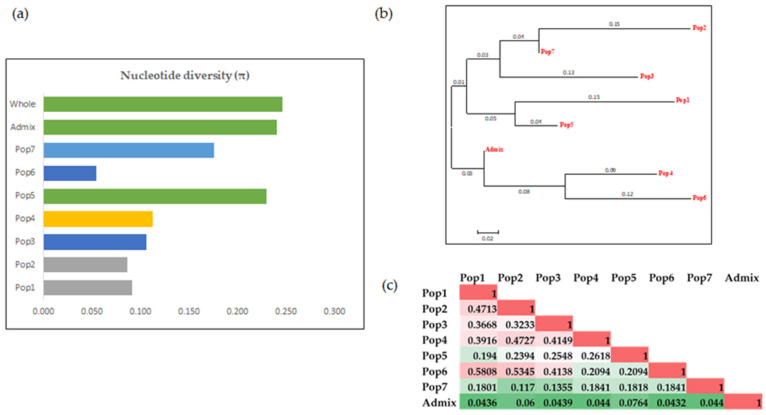
The genetic diversity analysis. The genetic diversity based on (**a**) the nucleotide diversity per site (π), (**b**) the fixation index (Fst), and (**c**) the matrix showing the pairwise Fst values between the populations.

The genetic diversity among the subpopulations identified by the structure analysis and PCoA was computed using the average nucleotide diversity (π) and fixation index (Fst). The highest average π was observed for the whole population (0.246), followed by the admixture group and Pop5. The lowest genetic diversity was found for Pop6 (Figure 4a). We also estimated the average π per site (0.25) across the various chromosomes, which ranged from 0.27 (chromosome 8) to 0.23 (chromosomes 14 and 22), to understand the genome-wide bottleneck effects and genetic diversity (Supplementary Figure S2). Based on the Fst values, Pop2, Pop3, and Pop7 were in the same cluster, indicating their genetic relatedness in agreement with the PCoA. Pop1 and Pop5 were clustered separately whereas Pop4, Pop6, and the admixture were clustered together. Although the nucleotide diversity showed that Pop1 and Pop2 had almost similar π values (0.091 for Pop1 and 0.086 for Pop2), they were grouped into two different clusters, indicating that they were genetically distant but had less variability. Similarly, Pop3 and Pop4 had similar π values but were genetically diverse (Figure 4).

#### 3.3.2. Cluster Analysis

**Figure 5:**
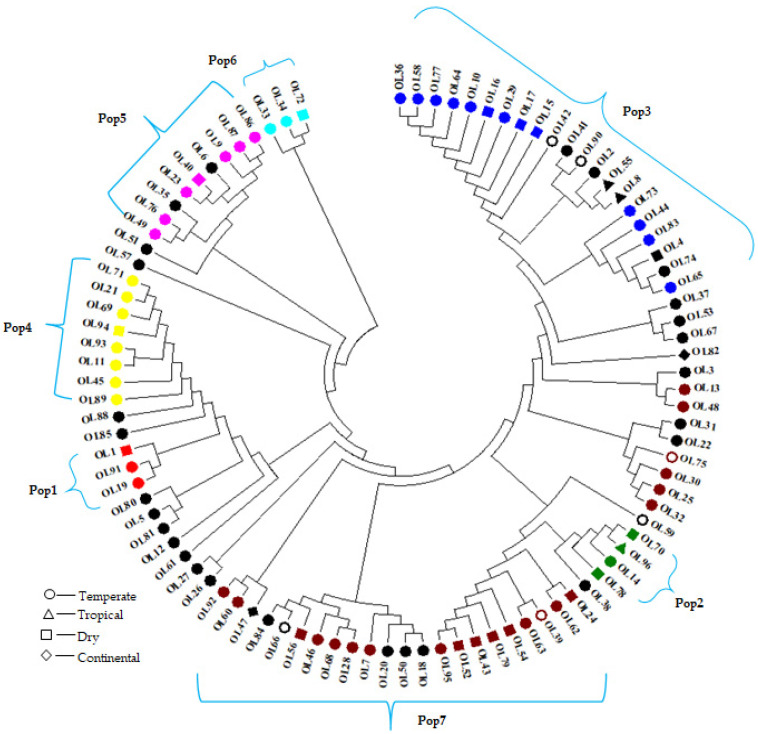
Phylogenetic analyses of the 96 olive cultivars using the neighbor-joining method. Different colors depict the structure analysis generated populations. Legends indicate the climatic zones from where the accessions were originated. Colors represent different subpopulations of the germplasm; red color = subpopulation1, green = subpopulation2, blue = subpopulation3, yellow = subpopulation4, pink = subpopulation5, cyan = subpopulation6, maroon = subpopulation7, black = admixture group.

We conducted a genotype-based phylogenetic analysis using the neighbor-joining method implemented in MEGA7 [41] The genotype-based cluster analysis reflected a similar population structure, resulting in seven distinct clusters but not aligning with the climatic zones. For example, all the accessions from the dry and temperate zones were clustered across all subpopulations. Among the three tropical accessions, one grouped in Pop2 and the remaining two were in the admixture. All Continental accessions were also grouped in the admixture (Figure 5).

#### 3.3.3. Analysis of Molecular Variance (AMOVA)

An AMOVA analysis was performed to understand the underlying sources of the genetic variation in the germplasm. When the accessions were divided based upon the population structure as of the first level of stratification, the results of the AMOVA indicated that the genetic variation mainly occurred within a population (67%) whereas 33% of the variation was attributed to the difference among populations (Table 3). However, when the climatic zone was included as a second level of stratification of the population structure to group the accessions, even though the majority of genetic variations arose from within the samples (70%, as shown in Table 3), the primary source of the genetic variation (26%) was the climatic zone within a population than the among the population variation (4%) (Table 4).

### 3.4. Marker Characteristics across the Populations

**Figure 6:**
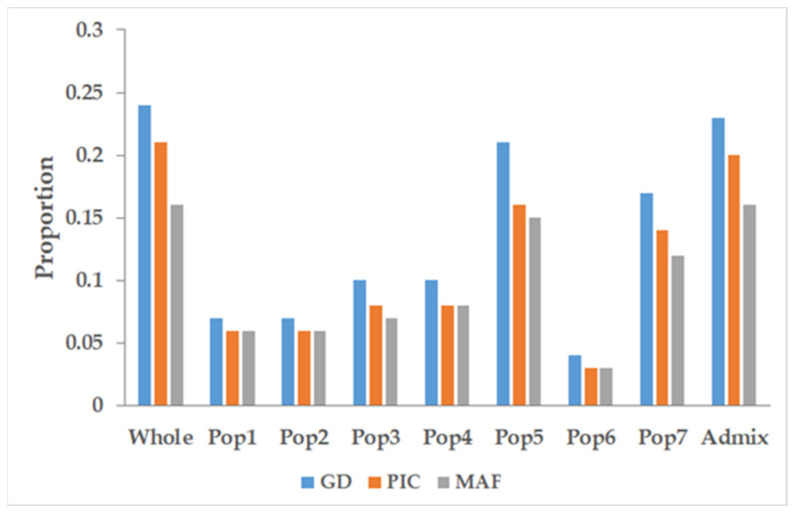
Population-wide marker characteristics. The marker characteristics in terms of the gene diversity (GD), polymorphic information content (PIC), and minor allele frequency (MAF) across the seven subpopulations and the whole population.

For the minor allele frequency (MAF), gene diversity (GD), and polymorphic information content (PIC), the admixture group had the highest MAF, GD, and PIC values overall, followed by Pop5 and Pop7, among the populations. In contrast, Pop6 had the lowest value for each of the characteristics. The remaining Pop1, Pop2, Pop3, and Pop4 had almost similar MAF, GD, and PIC values (Figure 6).

## 4. Discussion

### 4.1. GBS Analysis of the Olive Genomes

Knowing the genetic variability in the collection of the available pre-adapted olive genotypes is a prerequisite for the US olive improvement program. Despite the availability of genetic studies of numerous European or Mediterranean germplasm accessions, little is known about the population structure or genetic diversity of the existing USDA collection of olives. An accurate molecular documentation is critical for germplasm curators, breeders, and geneticists as well as plant pathologists. The olive germplasm collections at several centers have been largely influenced by natural dissemination and human migration as well as multilocal selection, breeding, and propagation [29,44,45]. The genetic structure of the USDA germplasm collection consisting of 110 olive cultivars characterized using fifteen microsatellite SSRs markers [29] showed a significant diversity but low levels of differentiation among the olive cultivars within this collection. We chose to use SNPs to distinguish the germplasm collection because of the advent of next-generation sequencing technologies and genome-wide screening capabilities [5]. GBS has become the most popular SNP discovery and genotyping technique in plant species [46,47]. So far, a few studies have used the GBS technique to understand the genetic diversity of local collections of olive cultivars, leading to regional olive improvement programs [10,11,48]. A genome-wide association study (GWAS) successfully used GBS markers to map five agronomic traits using a collection of olive accessions [3]. A recent study involving 57 olive cultivars of European and Mediterranean origins showed that GBS-SNP loci effectively corrected the relationship among different cultivars, further confirming the utility of GBS markers for genetic diversity analyses [5]. Here, we performed a molecular characterization of 96 olive genotypes from the USDA core collection, including six regionally popular varieties using GBS-generated SNP markers. The accessions represented diverse cultivars from *Olea europaea* L. originating from 18 countries across four climatic zones.

### 4.2. Features of the SNP Markers

The average number of 3.99 million sequence reads per sample obtained in this study was much higher than other similar studies of olives [10,11]. We obtained 54,075 high-quality SNPs after filtering, which was much higher than previous studies of olives [3,11,49]. On the contrary, these numbers were smaller than a study [5] involving 57 olive cultivars. The results obtained in this study were mainly due to different sources of olive collections and the platform used to resolve the amplified products. However, unlike previous studies, we used an improved reference genome of *Olea europaea* cv.; “Farga” (version Oe9) [3] was developed by anchoring the previously used Oe6 version [48] to a publicly available genetic map [11].

Transition SNPs were more available than transversions, consistent with the previous studies of olives and other plant species [5,19,49,50,51]. As parameters for describing the variability of the SNP marker, the minor allele frequency, gene diversity (GD), and polymorphic information content (PIC) were used in the study. Understanding the GD and PIC values were critical to finding the polymorphisms among the accessions, selection pressure on the allele, and locus mutation rate over the period [19]. In the current study, the average GD and PIC values were 0.244 and 0.206, respectively. The PIC value of the SNP marker was not equal or close to the GD because of its biallelic nature that restricts an allele frequency increase [52]. Most PIC values were below 50%, consistent with previous studies of olives [5,53]. A total of 33% of the SNPs of this study had a PIC value > 0.25, which was half of its maximum theoretical PIC value (0.5) [54]. Hence, one third of the identified SNPs in this study that showed a 50% resolution ability over its maximum capacity could help in marker-assisted selections or breeding for developing new varieties.

### 4.3. Features of the US Repository Olive Population

The population structure analysis revealed seven subpopulations (K = 7) with admixture groups in the US olive germplasm collection based on a 0.70 membership probability. This analysis coincided well with the PCoA and genotype-based phylogenetic analysis. The structure analysis outcomes of the study were similar to a previous study [3], which identified six subpopulations in a subset of the US collection of olives. The Bayesian and distance-based clustering of the accessions in this study did not align with the geographical origin and climatic zones, suggesting that the US accessions collected from different countries and climatic zones were genetically similar, possibly because of a limited selection and crossing during early domestication [3]. Although this is in agreement with the geographic origins was consistent with previous GBS-based studies of olives [3,5,55], it was contrary to a report that used a subset of SSR markers to show a partial clustering of the US repository accessions with the geographical origin [56,57]. The difference may be due to a significantly lower number of markers used to make the conclusions described in the SSR study. Nonetheless, a low level of genetic differentiation was consistent with little diversity among the clusters identified by the population structure and phylogenetic analysis in our study. Further, in our study, two major groups with subgroups based on Fst values had a low genetic divergence among the clusters.

The US accessions were grouped into Pop1, 3, 4, 6, and 7 as well as the admixture group. Interestingly, most accessions of these subpopulations were from temperate and dry climatic zones, signifying the restrictive selection of the accessions in these regions. Similarly, the accessions from the non-native areas (Russia, Japan, and Peru) were grouped into the admixture groups with accessions from the Mediterranean regions (e.g., Cyprus, Greece, Italy, Spain, and France). It may be due to a shared ancestry, outcrossing genotypes from diverse backgrounds, or selection pressure to adapt to the local environment.

The Fst values describe the genetic differentiation between two subpopulations [3]. This study observed significant differences among the seven subpopulations except between Pop7 vs. Pop2 and Pop7 vs. Pop3, which was consistent with the phylogenetic analysis and PCoA where the genotypes were grouped based on the population. The pairwise Fst values between most of the population groups were higher than 0.15, suggesting a high genetic differentiation in the US repository collection. These results also supported the AMOVA analysis, which showed that a significant (*p* ≤ 0.001) genetic variation existed among and within the subpopulations. Although most of the genetic variation (67%) came from within the population, among the subpopulations was the source of a 33% genetic variation. After including the climatic zone as a sub-source of the genetic variation, the climatic zones within the subpopulations accounted for 26 and 4% of the genetic variation among the populations. In both AMOVA analyses, the genetic variation within the subpopulations was the highest and the geographical location also explained a major source of genetic variability. It is believed that the domestication of the olive started 6000 years ago in the eastern Mediterranean basin, from where it migrated to different regions across the world during classical civilization [34,56]. The selection pressure for quality and productivity traits alongside adaptation to a particular climatic area may have contributed towards the variation by geographic locations. The low level of diversity among the subpopulations was consistent with studies of other plants where the among population contributed to the low amount of genetic diversity [19,55]. In terms of allelic patterns and genetic diversity within subpopulations or clusters, Pop5 and Pop7 were more genetically diverse—as reflected by the higher gene and nucleotide diversity values than the other remaining groups, possibly because: (1) the ancestor accessions of these groups were more genetically diverse; (2) there was a selective crossing or natural outcrossings with genotypically diverse cultivars; and (3) there was a selection by the environmental conditions to be more diverse. This information is essential for parent selection in the US olive improvement program to maintain and monitor the genetic diversity required for a successful breeding program or to broaden the genetic basis of the US olive germplasm.

## 5. Conclusions

This study used high-throughput GBS technology for SNP genotyping to explore genetic diversity and structures in the olive accessions within the US repository. The study identified and examined the features of the SNP markers to facilitate future efforts of US olive genetic improvements. The SNP markers performed well in terms of the polymorphism, genetic diversity, and population structure analysis. A total of 33% of the total SNPs used in the study showed half of their maximum PIC value (0.5), indicating their suitability for future marker-assisted breeding. Overall, our germplasm was genetically diverse with seven subpopulations. This genetic diversity could be helpful for future breeding programs through the selection of suitable parents for developing new olive cultivars with desirable agronomical characteristics adapted to US climatic challenges. The seven subpopulations identified in this study did not align with the geographical origin or climate zones, possibly due to regional selection and domestication. The subpopulations Pop5 and Pop7were genetically diverse whereas Pop6 was less diverse. This information could be helpful to select parents from various groups to widen genetic diversity during olive improvement and breeding programs. The overall findings of this study will help to conduct genetic mapping, association mapping, genomic selection, and marker-assisted breeding.

## Figures and Tables

**Table 1:** List of the countries of origin of the 96 olive accessions with their corresponding climatic zone.

Origin		No. of Genotypes
Climatic zone	Country	
Continental	Russia	2
Dry	Algeria	1
	Argentina	1
	Chile	1
	Egypt	3
	Israel	1
	Morocco	1
	Pakistan	1
	Palestine	1
	Syria	1
	Tunisia	5
Temperate	Albania	2
	Cyprus	3
	France	5
	Greece	5
	Italy	22
	Japan	1
	Spain	14
	US	17
Tropical	Colombia	1
	Peru	2
Unknown		6

**Table 2:** Summary result of the SNP types.

	Transitions		Transversions			
Type of SNP	A/G	C/T	A/C	A/T	G/T	C/G
Number of sites	15,875	16,010	5513	7476	5288	3913
Frequencies	29.36%	29.61%	10.20%	13.83%	9.78%	7.24%
Total	31,885 (59.96%)		22,190 (41.04%)			

**Table 3:** Analysis of molecular variance (AMOVA) based on the population as being the source of the genetic variation.

Source of Variation	df	SS	MS	Variation	*p*-Value
Between the population	7	630,346.08	90,049.44	32.94	0.001
Within the population	88	1,277,390.84	14,515.80	67.06	0.001
Total	95	1,907,736.91	20,081.44	100.00	

**Table 4:** Analysis of molecular variance (AMOVA) based on the population and climatic zone as being the sources of the genetic variation.

Source of Variation	df	SS	MS	Variation	*p*-Value
Between the population	7	310,271.04	44,324.43	4.29	0.04
Between the climatic zones within the population	13	493,233.74	37,941.06	25.82	0.001
Within the population	75	1,104,232.13	14,723.10	69.89	0.001
Total	95	1,907,736.91	20,081.44	100.00	

## Data Availability

Data can be made available upon reasonable request.

## Supplementary Materials

The following are available online at https://www.mdpi.com/article/10.3390/genes12122007/s1: Table S1: Olive accessions with their corresponding USDA plant ID and population groups identified in the structure analysis. Table S2: GBS-generated sequencing reads per sample. Figure S1: Tassel GBS Pipeline Version 2. Figure S2: Nucleotide diversity per site (π) for each chromosome and scaffold.
