# Supplementary material for: Genetic Diversity and Population Structure Analysis of the USDA Olive Germplasm Using Genotyping-By-Sequencing (GBS)
Source: Genes (Basel). 2021 Dec 17;12(12):2007. doi: 10.3390/genes12122007 (PMC8701156; doi:10.3390/genes12122007)
Supplement: Supplementary file 1 [file genes-12-02007-s001.zip › genes-1505491-supplementary.pdf]

Table S1. Olive accessions with their corresponding USDA plant ID and population groups identified in structure analysis

ID	Pop	Plant ID	Plant name	Origin
OL1	Pop1	DOLE 125	Cucca	Argentina
OL10	Pop3	DOLE 14	Mission	US
OL11	Pop4	DOLE 181	Frantoio	Albania
OL12	Admix	DOLE 188	Oblonga Seedling	US
OL13	Pop7	DOLE 122	Piconia	US
OL14	Pop2	DOLE 163	No. 1 Sevillano	Cyprus
OL15	Pop3	DOLE 36	Toffahi of Egypt	Egypt
OL16	Pop3	DOLE 24	Sigoise	Algeria
OL17	Pop3	DOLE 84	Dolce del Marocco	Morocco
OL18	Admix	DOLE 34	Mission	US
OL19	Pop1	DOLE 51	Columello	France
OL2	Admix	DOLE 70	Cordovil	Italy
OL20	Admix	DOLE 26	Cucca	Italy
OL21	Pop4	DOLE 38	Columello	US
OL22	Admix	DOLE 3	Oblonga	France
OL23	Pop5	DOLE 174	Ascolana Dura	Italy
OL24	Pop7	DOLE 50	Liguria de Catamarca	Chile
OL25	Pop7	DOLE 152	Karolia	Greece
OL26	Admix	DOLE 88	Ascolana Tenera	US
OL27	Admix	DOLE 91	Chetoui	US
OL28	Pop7	DOLE 123	Campanil	US
OL29	Pop3	DOLE 46	Mission	US
OL3	Admix	DOLE 31	Dwarf D	US
OL30	Pop7	DOLE 28	Ascolana Tenera	Italy
OL31	Admix	DOLE 54	Ascolana Dura	Cyprus
OL32	Pop7	DOLE 10	Ascolana Tenera	Italy
OL33	Pop6	DOLE 106	Bouquetier	Italy
OL34	Pop6	DOLE 116	#219	US
OL35	Admix	DOLE 186	Arbussana	Spain
OL36	Pop3	DOLE 55	Mission	Spain
OL37	Admix	DOLE 13	Gordal Sevillana	Spain
OL38	Admix	DOLE 29	Oliva de Cerignola	Greece

OL39	Pop7	DOLE 80	Karydolia	Unknown
OL4	Admix	DOLE 58	Manzanilla de Sevilla	Tunisia
OL40	Pop5	DOLE 89	Chemlali di Stax	Tunisia
OL41	Admix	DOLE 73	Picual	Spain
OL42	Admix	DOLE 101	3S5-117	Unknown
OL43	Pop7	Dole 15	Meski	Tunisia
OL44	Pop3	DOLE 52	Manzanilla de Sevilla	Spain
OL45	Pop4	DOLE 76	Bouteillon	US
OL46	Pop7	DOLE 170	Rigali	Italy
OL47	Admix	DOLE 183	Nikitskaya Krupnoplodn	Russia
OL48	Pop7	DOLE 118	Uovo di Piccione	Italy
OL49	Pop5	DOLE 39	Arbequina	Spain
OL5	Admix	DOLE 27	Leccino	Italy
OL50	Admix	DOLE 202	Cayet Roux	France
OL51	Admix	DOLE 108	Black Italian	Italy
OL52	Pop7	DOLE 83	Meski	Tunisia
OL53	Admix	DOLE 142	Lechin de Sevilla	Spain
OL54	Pop7	DOLE 161	Toffahi of Syria	Syria
OL55	Admix	DOLE 86	Azapa	Peru
OL56	Pop7	DOLE 23	Souri	Palestine
OL57	Admix	DOLE 214	Frantoio	Italy
OL58	Pop3	DOLE 140	Mission	US
OL59	Admix	DOLE 162	Obliza	Unknown
OL6	Admix	DOLE 71	Redding Picholine	Italy
OL60	Pop7	DOLE 203	Aglandau	France
OL61	Admix	DOLE 19	San Francesco	Italy
OL62	Pop7	DOLE 185	Thrombolea	Greece
OL63	Pop7	DOLE 180	Mixani	Albania
OL64	Pop3	DOLE 45	Mission	Italy
OL65	Pop3	DOLE 49	Manzanilla de Sevilla	Spain
OL66	Admix	DOLE 173	Late Blanquette	Unknown
OL67	Admix	DOLE 64	Lechin de Sevilla	Spain
OL68	Pop7	DOLE 169	NO. 65A	Cyprus
OL69	Pop4	DOLE 204	Cailletier	France
OL7	Pop7	DOLE 155	Prunara	Italy
OL70	Pop2	DOLE 171	Toffahi of Egypt	Egypt
OL71	Pop4	DOLE 17	Frantoio	US
OL72	Pop6	DOLE 176	880333	Pakistan

OL73	Pop3	DOLE 141	Manzanilla de Sevilla	Spain
OL74	Admix	DOLE 11	Manzanilla de Sevilla	Spain
OL75	Pop7	DOLE 156	UC 49-14 (Asoclano X Ba)	Unknown
OL76	Pop5	DOLE 111	Arbequina	Spain
OL77	Pop3	DOLE 99	Mission	US
OL78	Pop2	DOLE 145	Balady	Egypt
OL79	Pop7	DOLE 68	Meski	Tunisia
OL8	Admix	DOLE 8	Azapa	Peru
OL80	Admix	DOLE 143	Maurino	Italy
OL81	Admix	DOLE 184	Leccino	Italy
OL82	Admix	DOLE 182	Nikitskaya #1	Russia
OL83	Pop3	DOLE 77	Manzanilla de Sevilla	Spain
OL84	Admix	DOLE 103	Verdale	Japan
OL85	Admix	NA	Tosca	Italy
OL86	Pop5	NA	Koroneiki	Greece
OL87	Pop5	NA	Arbosana	Spain
OL88	Admix	NA	Pendolino	Italy
OL89	Pop4	NA	Coratina	Italy
OL9	Pop5	DOLE 1	Tragolea	Greece
OL90	Admix	NA	Hoji Blanca	Unknown
OL91	Pop1	DOLE 189	Kalamata	US
OL92	Pop7	DOLE 138	Grappolo	US
OL93	Pop4	DOLE 63	Vassailika	Italy
OL94	Pop4	DOLE 167	Merhavia	Israel
OL95	Pop7	DOLE 102	Verdeal	Italy
OL96	Pop2	DOLE 129	Azapa	Colombia

Table S2. GBS generated sequencing reads per sample

Sample ID	Demultiplexed Reads
OL1	3317866
OL10	3053302
OL11	3033185
OL12	3110072
OL13	2186603
OL14	3269442
OL15	2029644
OL16	1985963
OL17	2237227
OL18	2038640
OL19	2214458
OL2	2609196
OL20	2226781
OL21	2436145
OL22	1215771
OL23	2808998
OL24	2087637
OL25	1511558
OL26	2210748
OL27	2529926
OL28	2592658
OL29	2170077
OL3	2196400
OL30	2632664
OL31	2357842
OL32	2541308
OL33	1613726
OL34	1704443
OL35	3053990
OL36	2285077
OL37	1787461
OL38	2075315
OL39	2305757
OL4	2262265
OL40	2183796
OL41	2395033
OL42	2453127
OL43	1845754
OL44	2577312

OL45	2756697
OL46	2496683
OL47	2708100
OL48	2741441
OL49	2374163
OL5	2548155
OL50	2217464
OL51	2887932
OL52	2647649
OL53	2845336
OL54	3301932
OL55	2700558
OL56	2049412
OL57	2039939
OL58	3840527
OL59	2187293
OL6	3046136
OL60	3027538
OL61	2610463
OL62	206437
OL63	1643780
OL64	2990926
OL65	2682781
OL66	7787896
OL67	10203610
OL68	9021471
OL69	8109989
OL7	2450816
OL70	6383413
OL71	9340618
OL72	5485789
OL73	8591809
OL74	8612153
OL75	11040211
OL76	8522280
OL77	7567117
OL78	6505804
OL79	7074726
OL8	3223964
OL80	6328315
OL81	2403946
OL82	8102452
OL83	11352205

OL84	9204732
OL85	10394819
OL86	9259855
OL87	9081089
OL88	9668377
OL89	9476735
OL9	3505804
OL90	8477952
OL91	7851648
OL92	10357789
OL93	9900149
OL94	10283001
OL95	6968596
OL96	6539299

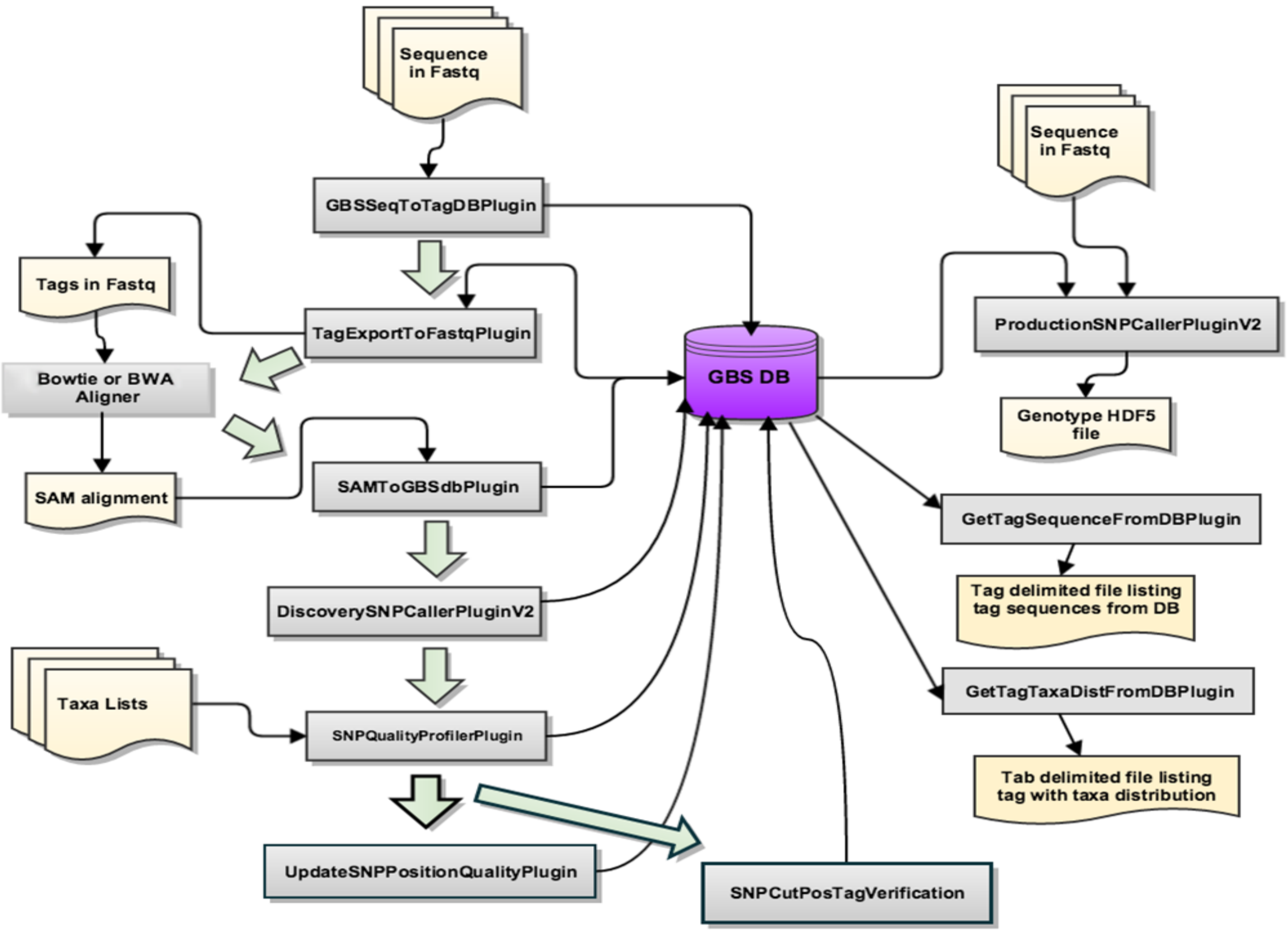

Figure S1. Tassel GBS Pipeline Version 2 (UW Bioinformatics Resource Center Tassel v2 pipeline GBS report (wisc.edu)

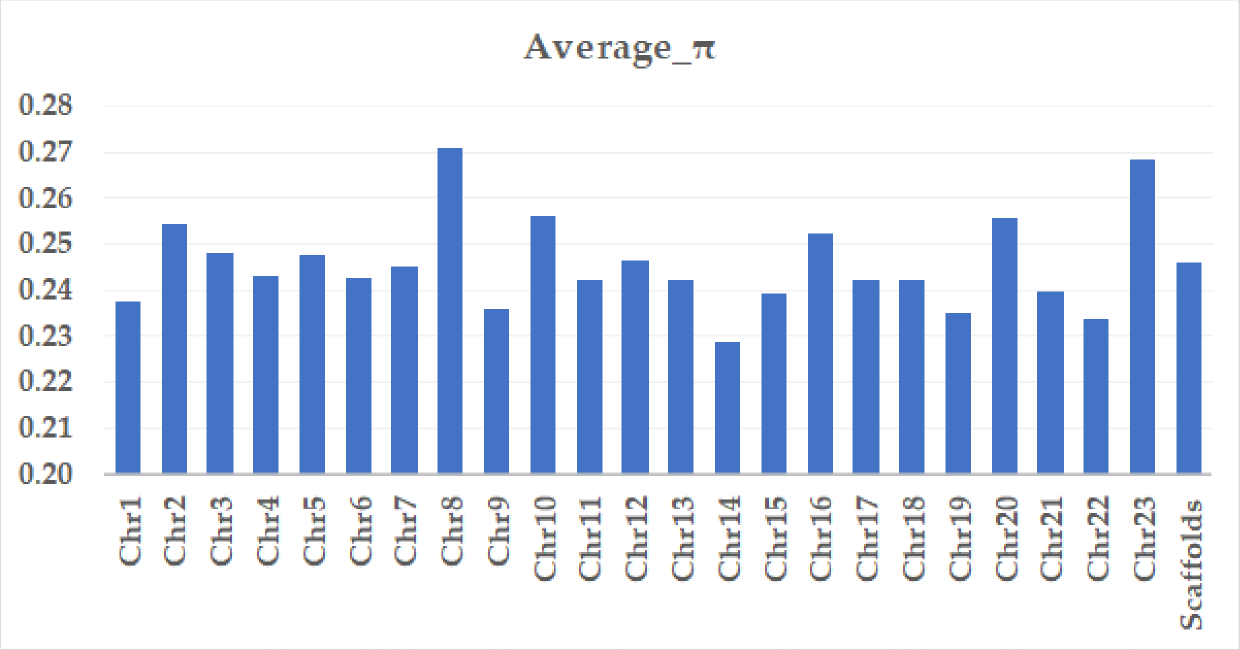

Figure S2. Nucleotide diversity per site (π) for each chromosome and scaffolds.
